# Whole Genome Sequencing Identifies Novel Mutations Associated With Bedaquiline Resistance in *Mycobacterium tuberculosis*

**DOI:** 10.3389/fcimb.2022.807095

**Published:** 2022-05-27

**Authors:** Qinglong Guo, Jing Bi, Qiao Lin, Taosheng Ye, Zhongyuan Wang, Zhaoqin Wang, Lei Liu, Guoliang Zhang

**Affiliations:** ^1^ National Clinical Research Center for Infectious Diseases, Guangdong Provincial Clinical Research Center for Tuberculosis, Shenzhen Third People’s Hospital, Southern University of Science and Technology, Shenzhen, China; ^2^ Department of Traditional Chinese Medicine, The Baoan People’s Hospital of Shenzhen, Shenzhen University, Shenzhen, China

**Keywords:** *Mycobacterium tuberculosis*, bedaquiline, drug resistance, *rv0678*, *glpK*

## Abstract

Bedaquiline (BDQ), a new antitubercular agent, has been used to treat drug-resistant tuberculosis (TB). Although mutations in *atpE*, *rv0678*, and *pepQ* confer major resistance to BDQ, the mechanisms of resistance to BDQ *in vitro* and in clinical settings have not been fully elucidated. We selected BDQ-resistant mutants from 7H10 agar plates containing 0.5 mg/L BDQ (the critical concentration) and identified mutations associated with BDQ resistance through whole genome sequencing and Sanger sequencing. A total of 1,025 mutants were resistant to BDQ. We randomly selected 168 mutants for further analysis and discovered that 157/168 BDQ-resistant mutants harbored mutations in *rv0678*, which encodes a transcriptional regulator that represses the expression of the efflux pump, MmpS5–MmpL5. Moreover, we found two mutations with high frequency in *rv0678* at nucleotide positions 286–287 (CG286–287 insertion; accounting for 26.8% [45/168]) and 198–199 (G198, G199 insertion, and G198 deletion; accounting for 14.3% [24/168]). The other mutations were dispersed covering the entire *rv0678* gene. Moreover, we found that one new gene, *glpK*, harbors a G572 insertion; this mutation has a high prevalence (85.7%; 144/168) in the isolated mutants, and the minimum inhibitory concentration (MIC) assay demonstrated that it is closely associated with BDQ resistance. In summary, we characterized 168/1,025 mutants resistant to BDQ and found that mutations in *rv0678* confer the primary mechanism of BDQ resistance. Moreover, we identified a new gene (*glpK*) involved in BDQ resistance. Our study offers new insights and valuable information that will contribute to rapid identification of BDQ-resistant isolates in clinical settings.

## Introduction

Multidrug-resistant tuberculosis (MDR-TB), caused by drug-resistant *Mycobacterium tuberculosis* (Mtb), results from various factors, including substandard treatment regimens, treatment nonadherence, drug malabsorption, and drug–drug interactions during anti-TB therapy. This hampers the efforts of the World Health Organization (WHO)’s End TB Strategy (Nguyen et al., 2018; World Health Organization, 2019). In 2018, MDR-TB including rifampicin-resistant TB accounted for 3.4% of new TB cases and 18% of previously treated cases (World Health Organization, 2019). Moreover, approximately 6.2% of MDR-TB cases develop extensively drug-resistant TB (World Health Organization, 2019). Therefore, there is an urgent need to discover new and effective anti-TB drugs.

Bedaquiline (BDQ), a new antitubercular agent, has better efficacy against both drug-susceptible and drug-resistant Mtb *in vitro* (Andries et al., 2005) and improved treatment outcomes and cure rates among patients with drug-resistant TB (Diacon et al., 2014; Schnippel et al., 2018). As a result, WHO recommended the addition of BDQ to treatment regimens for drug-resistant TB (WHO, 2019). However, since the introduction of BDQ to treatment regimens, BDQ resistance has also emerged (Veziris et al., 2017).

BDQ exerts its antitubercular activity by inhibiting the activity of the F_1_F_0_-adenosine triphosphate (ATP) synthase encoded by the *atpE* gene (Andries et al., 2005). Therefore, mutations in *atpE* (A28V, A28P, G61A, A63P, and I66M) are associated with high levels of BDQ resistance (10–128-fold MIC). BDQ-resistant strains are currently being selected in *in vitro* studies, but they are rarely found in patients with TB (Nieto Ramirez et al., 2020), thus suggesting that mutations in the *atpE* gene have a fitness cost within these patients in comparison to *in vitro* cultures. Moreover, there is another ATP-synthase-independent mechanism of BDQ resistance (Nguyen et al., 2018). The drug efflux system functions as a non-target-based mechanism related to resistance against many antimicrobials, including BDQ (Andries et al., 2014). The *rv0678* gene encodes the Mtb transcriptional repressor of the MmpS5–MmpL5 efflux system, and high frequency mutations in the *rv0678* gene result in low BDQ resistance (2–8-fold MIC) *in vitro* and in clinical settings (Hartkoorn et al., 2014; Coeck et al., 2015). Mutations in the *rv0678* gene result in damaged function and upregulation of the MmpS5–MmpL5 efflux system. Interestingly, the MmpS5–MmpL5 efflux pump-based mechanism also exists in clofazimine resistance (Hartkoorn et al., 2014).

A previous study showed that *pepQ* gene mutations also lead to low-level BDQ resistance (up to fourfold) in mice (Almeida et al., 2016; Degiacomi et al., 2020) To our knowledge, mutations in atpE, rv0678, and pepQ gene have shown to be the major mechanisms of Mtb resistant to BDQ. However, Mtb isolates from one patient showed an increased MIC to BDQ, but no mutations were found in *atpE*, *Rv0678*, and *pepQ* and their respective upstream regions (Andres et al., 2020), suggesting the existence of other undetermined mechanisms of BDQ resistance. Consistent with this, Peterson et al. found that BDQ-treated Mtb could initiate a regulatory network regulated by Rv0324 and Rv0880 that coordinates multiple resistance mechanisms to push Mtb into a BDQ-tolerant state (Peterson et al., 2016). Therefore, the identification of new BDQ resistance-related genes will contribute to a comprehensive understanding of BDQ-resistant mechanisms that are critical for reducing the emergence of resistance and anti-TB drug discovery.

This study investigated BDQ-resistance-related mutations through Sanger and whole genome sequencing (WGS).

## Materials and Methods

### Bacterial Strains and Culture Conditions

The Mtb H37Rv strain was cultured in Middlebrook 7H9 broth (7H9; Difco, Detroit) liquid medium containing 0.05% Tween 80 and 10% oleic acid, albumin, dextrose, and catalase (OADC) or plated on Middlebrook 7H10 agar medium (Difco, Detroit) containing 10% OADC and 0.5% glycerol.

### BDQ-Resistant Mutant Selection

BDQ-resistant mutants were screened, as previously shown (Zhang et al., 2015; Pi et al., 2019). In brief, log phase Mtb H37Rv cultures were spread on 7H10 agar plates inoculated with 0.5 mg/L BDQ (Abcam, Cambridge, UK) and incubated at 37°C for 4 weeks. The critical concentration, which is approximately eightfold MIC, was chosen based on the MIC values reported in previous studies: clinical BDQ-resistant isolates have MIC values >0.25 mg/L (Veziris et al., 2017), and low-level BDQ resistant strains (with 2–8-fold MIC) are often seen both *in vitro* and in clinical settings; therefore, 0.5 mg/L was used for mutant selection. All colonies were picked and transferred onto new Middlebrook 7H10 agar plates containing 0.5 mg/L BDQ to demonstrate their resistance.

### Gene Mutation Analysis Through WGS

Genomic DNA extracted from BDQ-resistant strains and Mtb H37Rv using cetyltrimethylammonium bromide (Benjak et al., 2015) was analyzed by WGS using the Illumina MiSeq platform (Illumina Inc., San Diego, CA, USA) (Zhang et al., 2015). For each mutant, approximately 500 Mb to 1.5 Gb (110- to 350-fold genome coverage) sequences were produced, followed by the removal of the barcodes. Single-nucleotide variants (SNVs), insertions, and deletions harboring 1–58 bp were sorted and called with more than four reads using the Mtb H37Rv genome (NC_000962.3) as a reference. The proline–glutamic acid/proline–proline–glutamic acid family genes with mutations were not included in the analysis. Mutations harbored by MtbH37Rv contrasting to the online genome (NC_000962.3) were also removed and not analyzed.

### PCR and Sanger Sequencing

The BDQ-resistance-related genes were subjected to PCR amplification using the primers listed in **Supplementary Table S1** and the genomic DNA of BDQ-resistant isolates selected *in vitro* as a template. The obtained PCR products were sent for sequencing using the Sanger method to determine the mutations harbored in these genes in the isolated BDQ-resistant mutants.

### Antimicrobial Susceptibility Testing

The indicated mutants were picked from 7H10 agar plates in a tube containing 2 ml 7H9 liquid medium containing 10% OADC. After dispersing with a BACspreader (TB Healthcare, Guangdong, China) for 1 min, the bacteria suspension was prepared. BDQ was double diluted in 7H9 liquid medium containing 10% OADC (20–0.078 mg/L). One hundred microliters of each dilution and 100 μl of the suspension were then mixed in a 96-well plate. MICs were shown as the lowest concentration of BDQ at which no visible growth was observed.

### Data Availability

The WGS data were submitted to the Sequence Read Archive of the National Center for Biotechnology Information (PRJNA766993).

## Results

### Selection of BDQ-Resistant Isolates

In order to select mutants resistant to BDQ, approximately 5×10^9^ bacteria were spread onto 7H10 agar plates containing 0.5 mg/L BDQ as numerated by the colony-forming units count in the absence of antibiotics. A total of 1,025 BDQ-resistant isolates were selected, and 168 mutants were randomly selected from the plates and sent for WGS (101 mutants) and PCR (67 mutants) analysis. The mutation frequency of BDQ-resistant mutants was approximately 2×10^−7^.

### WGS Identification of BDQ Resistance-Related Mutations

To determine the possible mechanisms of resistance to BDQ, the BDQ-resistant mutants and the parent strain H37Rv were performed WGS and genome sequence comparisons. As shown in **Table 1**, most of the BDQ-resistant mutants (94/101) had *rv0678* gene mutations, including SNVs, deletions, insertions, and deletion and insertion. Insertion and deletion mutations in the *rv0678* gene cause a frameshift and may result in the loss of its function. No BDQ resistance mutations were identified in the *atpE* and *pepQ* genes in these mutants (**Table 1**). Given that the WGS results suggested that mutations in *rv0678* conferred the primary resistance mechanism against BDQ for the mutants identified, PCR and Sanger sequencing of the *rv0678* gene were performed on a different set of 67 mutants (**Table 2**). Similar results were observed, and 63/67 mutants with *rv0678* gene mutations (including SNVs, deletion, and insertion) were identified (**Table 2**). In accordance with its function, which acts as a repressor of the MmpS5–MmpL5 efflux pump, these results reveal that the r*v0678* mutation leads to primary resistance to BDQ.

**Table 1:** Mutation analysis of 101 BDQ-resistant mutants of Mtb by whole-genome sequence analysis.

Gene	Mutation type	Nucleotide change	Amino acid change	Mutant count
*rv0678*	nonsynonymous SNV	T124G	W42G	1
*rv0678*	nonsynonymous SNV	T78G	Y26stop	1
*rv0678*	nonsynonymous SNV	T131C	L44P	1
*rv0678*	nonsynonymous SNV	T425G	L142R	8
*rv0678*	nonsynonymous SNV	G120T	L40F	1
*rv0678*	nonsynonymous SNV	G308C	G103A	1
*rv0678*	nonsynonymous SNV	C214T	R72W	2
*rv0678*	nonsynonymous SNV	T254A	V85D	1
*rv0678*	nonsynonymous SNV	T179C	L60P	5
*rv0678*	nonsynonymous SNV	G71T	G24V	1
*rv0678*	nonsynonymous SNV	T341C	L114P	1
*rv0678*	deletion	G184 deletion	62 codon shift	1
*rv0678*	deletion	AT436-437 deletion	146 codon shift	1
*rv0678*	deletion	G363 deletion	122 codon shift	3
*rv0678*	deletion	TGTTGGCATATATGGAGAACGTCGTCTCCGACGCCCTGGGGCGATACAGCCAGCGAAC425-482 deletion	142 codon shift	1
*rv0678*	deletion	A205 deletion	69 codon shift	1
*rv0678*	deletion	AGCAGCGGGGGGATCAG187-203 deletion	63 codon shift	1
*rv0678*	deletion	A209 deletion	70 codon shift	1
*rv0678*	deletion	C471 deletion	157 codon shift	1
*rv0678*	deletion	G216 deletion	73 codon shift	2
*rv0678*	deletion	T451 deletion	151 codon shift	2
*rv0678*	deletion	G198 deletion	67 codon shift	5
*rv0678*	deletion	C349 deletion	117 codon shift	1
*rv0678*	deletion	C158 deletion	53 codon shift	1
*rv0678*	deletion	CTGTT424-428 deletion	142 codon shift	1
*rv0678*	deletion	T95 deletion	32 codon shift	1
*rv0678*	deletion	A415 deletion	139 codon shift	1
*rv0678*	deletion	G19 deletion	7 codon shift	1
*rv0678*	deletion	GGA51-53 deletion	17 codon shift	1
*rv0678*	insertion	G439 insertion	147 codon shift	1
*rv0678*	insertion	CG286-287 insertion	97 codon shift	24
*rv0678*	insertion	G198 insertion	67 codon shift	10
*rv0678*	insertion	GAC346-348 insertion	D116 insertion	5
*rv0678*	insertion	G253 insertion	85 codon shift	1
*rv0678*	insertion	G430 insertion	144 codon shift	1
*rv0678*	insertion	A209 insertion	70 codon shift	1
*rv0678*	insertion	A275 insertion	92 codon shift	1
*rv0678*	deletion and insertion	CC279-280 deletion and TT279–280 insertion	R94W	1
*rv0678*	WT	\	\	7

**Table 2:** Mutation analysis of 67 BDQ-resistant mutants of Mtb by PCR and Sanger analysis.

Locus_tag_1	Nucleotide change	Amino acid change	Locus_tag_2	Nucleotide change	Amino acid change	Mutantcount
*rv0678*	C214T	R72W	*glpK*	G572 insertion	192 codon shift	1
*rv0678*	C214T	R72W	*glpK*	WT	WT	1
*rv0678*	T179C	L60P	*glpK*	G572 insertion	192 codon shift	3
*rv0678*	T341C	L114P	*glpK*	G572 insertion	192 codon shift	1
*rv0678*	T341C	L114P	*glpK*	WT	WT	1
*rv0678*	T350C	L117P	*glpK*	G572 insertion	192 codon shift	1
*rv0678*	G194A	G65E	*glpK*	G572 insertion	192 codon shift	1
*rv0678*	G304A	A102T	*glpK*	G572 insertion	192 codon shift	1
*rv0678*	C15G	D5E	*glpK*	G572 insertion and C814 deletion and C818 insertion	192 codon shift and 272 codon shift and 273 codon shift	1
*rv0678*	G269C	R90P	*glpK*	G572 insertion	192 codon shift	1
*rv0678*	G404A	R135Q	*glpK*	G572 insertion	192 codon shift	1
*rv0678*	G73A	G25S	*glpK*	G572 insertion	192 codon shift	1
*rv0678*	T365C	L122P	*glpK*	G572 insertion	192 codon shift	2
*rv0678*	A476C	Q159P	*glpK*	G572 insertion	192 codon shift	1
*rv0678*	G448C	V150P	*glpK*	G572 insertion	192 codon shift	1
*rv0678*	C343T	Q115M	*glpK*	G572 insertion	192 codon shift	1
*rv0678*	T461C	L154P	*glpK*	G572 insertion	192 codon shift	1
*rv0678*	T425G	L142R	*glpK*	G572 insertion	192 codon shift	1
*rv0678*	T425G	L142R	*glpK*	WT	WT	1
*rv0678*	T179G	L60R	*glpK*	G572 insertion and G646A	192 codon shift and E216K	1
*rv0678*	TGTTGGCATATATGGAGAACGTCGTCTCCGACGCCCTGGGGCGATACAGCCAGCGAAC425-482 deletion	142 codon shift	*glpK*	G572 insertion	192 codon shift	1
*rv0678*	G198 deletion	67 codon shift	*glpK*	G572 insertion	192 codon shift	6
*rv0678*	A53 deletion	19 codon shift	*glpK*	G572 insertion	192 codon shift	1
*rv0678*	C349 deletion	117 codon shift	*glpK*	G572 insertion	192 codon shift	1
*rv0678*	CG286-287 insertion	97 codon shift	*glpK*	G572 insertion	192 codon shift	20
*rv0678*	CG286-287 insertion	97 codon shift	*glpK*	WT	WT	1
*rv0678*	GAC346-348 insertion	D116 insertion	*glpK*	G572 insertion	192 codon shift	3
*rv0678*	G253 insertion	85 codon shift	*glpK*	G572 insertion	192 codon shift	1
*rv0678*	TCGATTGTTGGGC111-123 insertion	38 codon shift	*glpK*	G572 insertion	192 codon shift	1
*rv0678*	G199 insertion	67 codon shift	*glpK*	G572 insertion	192 codon shift	3
*rv0678*	G466 insertion	155 codon shift	*glpK*	G572 insertion	192 codon shift	1
*rv0678*	G39 insertion	13 codon shift	*glpK*	WT	WT	1
*rv0678*	WT	WT	*glpK*	G572 insertion	192 codon shift	3
*rv0678*	WT	WT	*glpK*	GG816-817 deletion and CC816-817 insertion	A273P	1

Among all the mutants subjected to WGS and Sanger sequencing analysis, we found two high frequency mutations in the *rv0678* gene. One hotspot was found at nucleotide positions 286–287 (CG286–287 insertion) and accounted for 26.8% (45/168) of the mutants (**Tables 1**, **2**). The second hotspot was found at nucleotide positions 198–199 (G198 deletion and insertion and G199 insertion) and accounted for 14.3% (24/168) of the mutants (**Tables 1**, **2**). The other mutations were widespread throughout the entire *rv0678* gene with no apparent clustering (**Tables 1**, **2**).

In addition, we identified a few new, previously undetermined genes that may be related to BDQ resistance. Those newly identified genes include *glpK*, *cobQ2*, *pitA*, *gid*, *rv2426c*, *rv2820c*, *rv3510c*, *rv0071*, *cya*, *rv2477c*, *lppB*, *rv0953c*, *rv2823c*, *rv0405*, *fhaA*, *rv2722*, *trcR*, *rv3785*, and *rv1723* (**Table 3**). Moreover, most *rv0678* mutants also harbored mutations in the newly identified genes, regardless of the *rv0678* mutation. In particular, we found highly prevalent mutations in *glpK* in the selected isolates, in addition to *rv0678* mutations (**Table 3**). Furthermore, our results showed that most BDQ-resistant mutants (86/101) with mutations in *rv0678* also harbored the G572 insertion mutation in *glpK* (**Table 3**). Interestingly, a proportion of BDQ-resistant mutants (6/101) with wild-type (WT) *rv0678* had a G572 insertion mutation in *glpK* (**Table 3**). PCR and Sanger sequencing analysis performed on the remaining 67 BDQ-resistant mutants showed similar results (58/67 mutant harboring *rv0678* mutations and the G572 insertion mutation in *glpK*; 3/67 harboring WT *rv0678* and G572 insertion mutation in *glpK*; **Table 2**). Therefore, it is likely that insertion mutations in the *glpK* gene are associated with BDQ resistance. To determine the relationship between the *glpK* gene mutation and BDQ resistance, an MIC assay was performed. *rv0678* (CG286–287 insertion, and A209, G198, and C348 deletions), *glpK* (G572 insertion), and *rv2820c* (A342T) mutants had the same MIC as that of mutants with WT *rv0678*, *glpK* (G572 insertion), and *rv2820c* (A342T) gene mutations (**Table 4**). Moreover, mutants harboring the WT *glpK* gene, *rv0678* (GC285–286 insertion), and *rv2820c* (A342T) gene mutations also showed the same MIC as mutants with WT *rv0678* gene, *glpK* (G572 insertion), and *rv2820c* (A342T) gene mutations, while the MIC of Mtb H37Rv was 0.078 µg/ml (**Table 4**). Together, these results suggest that insertion mutations in *glpK* may be responsible for resistance to BDQ.

**Table 3:** Hitchhiking mutations identified in BDQ-resistant mutants of Mtb.

Locus_tag_1	Locus_tag_2	Nucleotide change	Amino acid change	Locus_tag_3	Nucleotide change	Amino acid change	Mutantcount
*rv0678* W42G (1); 146 codon shift (1); WT (1);	*glpK*	C80G	T27S	*cobQ2* and *pitA*	G223A and CTGTGGGTGAT CGTGAG655-671 insertion	G75S and 226 codon shift	3
*rv0678* 122 codon shift (1);	*glpK*	C80G	T27S	*cobQ2* and *pitA* and *gid* and *rv2426c*	G223A and CTGTGGGTGATCGTGA G655-671 insertion and A362C and CT AGAGGAGACCCGATCGTG-17-3 deletion	G75S and 226 codon shift and E121A and M1 deletion	1
*rv0678* 122 codon shift (1);	*glpK*	C80G	T27S	*cobQ2* and *pitA* and *gid*	G223A and CTGTGGGTGATCGTGA G655-671 insertion and A362C	G75S and 226 codon shift and E121A	1
*rv0678* 147 codon shift (1)	*glpK*	G67A	G23S	*cobQ2* and *pitA* and *rv2820c* and *rv3510c*	G223A and CTGTGGGTGATCGTG AG655-671 insertion and A342T and G739A	G75S and 226 codon shift and K114N and D247N	1
*rv0678* 122 codon shift (1)	*glpK*	C80G	T27S	*cobQ2* and *pitA* and *gid* and *rv2820c*	G223A and CTGTGGGTGATCGTG AG655-671 insertion and A362C and A342T	G75S and 226 codon shift and E121A and K114N	1
*rv0678* 62 codon shift (1); Y26 stop (1);	*glpK*	A1036G	S346G	*cobQ* and *rv2820c* and *pitA*	G223A and A342T and CTGTGGGTGA TCGTGAG655-671 insertion	G75S and K114N and 226 codon shift	2
*rv0678* 97 codon shift (10); 67 codon shift (6); G103A (1); 157 codon shift (2); V85D (1); L60P (4); G24V (1); D116 insertion (3); WT (1); 53 codon shift (1); 151 codon shift (1); 139 codon shift (1); R72W (1)	*glpK*	G572 insertion	192 codon shift	*/*	/	/	33
*rv0678* 67 codon shift (1); 151 codon shift (1);	*glpK*	G572 insertion	192 codon shift	*rv0071*	C86T	A29V	2
*rv0678* 97 codon shift (3);	*glpK*	G572 insertion	192 codon shift	*cya*	T1070C	V357A	3
*rv0678* L44P (1); WT (1);	*glpK*	G572 insertion	192 codon shift	*cobQ2* and *pitA* and *rv2426c*	G223A and CTGTGGGTGATCGTGA G655-671 insertion and CTAGAGGA GACCCGATCGTG-17-3 deletion	G75S and 226 codon shift and M1 deletion	2
*rv0678* D116 insertion (1); 142 codon shift (1); 67 codon shift (1); 7 codon shift (1);	*glpK*	G572 insertion	192 codon shift	*rv2426c*	CTAGAGGAGACCCGATCGTG-17-3 deletion	M1 deletion	4
*rv0678* 97 codon shift (3); ME17_18 deletion and I insertion (1); 69 codon shift (1); WT (1); 67 codon shift (2); R72W (1); 70 codon shift (1);	*glpK*	G572 insertion	192 codon shift	*rv2820c*	A342T	K114N	10
*rv0678* L142R (4); 73 codon shift (1); 67 codon shift (1); L114P (1);	*glpK*	G572 insertion	192 codon shift	*cobQ2* and *pitA*	G223A and CTGTGGGTGATCGTG AG655-671 insertion	G75S and 226 codon shift	7
*rv0678* 97 codon shift (3);	*glpK*	G572 insertion	192 codon shift	*rv2820c* and *cya*	A342T and T1070C	K114N and V357A	3
*rv0678* 67 codon shift (1);	*glpK*	G572 insertion	192 codon shift	*cobQ* and *rv2820c* and *pitA*	G223A and A342T and CTGTGGGTG ATCGTGAG655-671 insertion	G75S and K114N and 226 codon shift	1
*rv0678* 97 codon shift (1);	*glpK*	G572 insertion	192 codon shift	*cya* and *rv2426c* and *rv2477c*	T1070C and CTAGAGGAGACCCGA TCGTG-17-3 deletion and A737G	V357A and M1 deletion and E246G	1
*rv0678* 63 codon shift (1);	*glpK*	G572 insertion	192 codon shift	*rv2820c* and *rv2426c*	A342T and CTAGAGGAGACCCGAT CGTG-17-3 deletion	K114N and M1 deletion	1
*rv0678* 97 codon shift (2);	*glpK*	G572 insertion	192 codon shift	*cya* and *lppB*	T1070C and C175A	V357A and H59N	2
*rv0678* L60P (1);	*glpK*	G572 insertion	192 codon shift	*rv0953c*	A643G	K215E	1
*rv0678* 117 codon shift (1); 85 codon shift (1);	*glpK*	G572 insertion	192 codon shift	*cobQ*	G223A	G75S	2
*rv0678* 97 codon shift (1);	*glpK*	G572 insertion	192 codon shift	*lppB*	A224G and C175A	Q75R and H59N	1
*rv0678* WT (3);	*glpK*	G572 insertion	192 codon shift	*lppB*	C175A	H59N	3
*rv0678* 144 codon shift (1);	*glpK*	G572 insertion	192 codon shift	*rv2823c*	TCGCCGACA304-312 insertion	IAD102-104 insertion	1
*rv0678* L142R (1);	*glpK*	G572 insertion	192 codon shift	*cobQ2* and *pitA* and *rv2823c*	G223A and CTGTGGGTGATCGT GAG655-671 insertion and TCGCCG ACA304-312 insertion	G75S and 226 codon shift and IAD102-104 insertion	1
*rv0678* L142R (1);	*glpK*	WT	WT	*cobQ*	G223A	G75S	1
*rv0678* L142R (1);	*glpK*	WT	WT	*cobQ* and *pitA*	G223A and CTGTGGGTGATCGTG AG655-671 insertion	G75S and 226 codon shift	1
*rv0678* R94W (1);	*glpK*	WT	WT	*cobQ* and *rv2426c*	G223A and CTAGAGGAGACCCGAT CGTG-17-3 deletion	G75S and M1 deletion	1
*rv0678* 142 codon shift (1);	*glpK*	G572 insertion	192 codon shift	*cobQ* and *pitA* and *lppB*	G223A and CTGTGGGTGATCGTG AG655-671 insertion and C175A	G75S and 226 codon shift and H59N	1
*rv0678* 32 codon shift (1);	*glpK*	G572 insertion	192 codon shift	*rv0405*	GC2275-2276 deletion	759 codon shift	1
*rv0678* 92 codon shift (1);	*glpK*	G572 insertion	192 codon shift	*cobQ* and *lppB*	G223A and C175A	G75S and H59N	1
*rv0678* 67 codon shift (1);	*glpK*	G572 insertion	192 codon shift	*cobQ2* and *pitA* and *fhaA*	G223A and CTGTGGGTGATCGTG AG655-671 insertion and TTACCCC GAGCAACGCGG729-746 deletion	G75S and 226 codon shift and Y-G 244-249 deletion	1
*rv0678* 70 codon shift (1);	*glpK*	G572 insertion	192 codon shift	*rv2722* and *lppB*	A126 deletion and C175A	42 codon shift and H59N	1
*rv0678* 67 codon shift (1);	*glpK*	G572 insertion	192 codon shift	*trcR* and *lppB*	C721G and C175A	P241A and H59N	1
*rv0678* 73 codon shift (1);	*glpK*	G572 insertion	192 codon shift	*cobQ* and *lppB* and *rv3785*	G223A and C175A and C629 insertion	G75S and H59N and 211 codon shift	1
*rv0678* L142R (1);	*glpK*	G572 insertion	192 codon shift	*cobQ2* and *pitA* and *rv1723*	G223A and CTGTGGGTGATCGTG AG655-671 insertion and C698T	G75S and 226 codon shift and P233L	1
*rv0678* L40F (1); D116 insertion (1); 67 codon shift (1);	*glpK*	WT	WT	WT	WT	WT	3

**Table 4:** MIC of BDQ-resistant mutants of Mtb.

Strain ID	Locus_tag_1	Locus_tag_2	Locus_tag_3	MIC of BDQ (mg/L)
65	*rv0678* [WT]	*glpK* [G572 insertion (192 codon shift)]	*rv2820c* [A342T (K114N)]	1.25
109	*rv0678* [WT]	*glpK* [G572 insertion (192 codon shift)]	*rv2820c* [A342T (K114N)]	1.25
113	*rv0678* [CG286-287 insertion (97 codon shift)]	*glpK* [WT]	*rv2820c* [A342T (K114N)]	1.25
48	*rv0678* [CG286-287 insertion (97 codon shift)]	*glpK* [G572 insertion (192 codon shift)]	*rv2820c* [A342T (K114N)]	1.25
75	*rv0678* [A209 deletion (70 codon shift)]	*glpK* [G572 insertion (192 codon shift)]	*rv2820c* [A342T (K114N)]	1.25
66	*rv0678* [G198 deletion (67 codon shift)]	*glpK* [G572 insertion (192 codon shift)]	*rv2820c* [A342T (K114N)]	1.25
110	*rv0678* [C348 deletion (117 codon shift)]	*glpK* [G572 insertion (192 codon shift)]	*rv2820c* [A342T (K114N)]	1.25
H37Rv	*rv0678* [WT]	*glpK* [WT]	*rv2820c* [WT]	0.078

## Discussion

In the present study, to better characterize the mechanisms of BDQ resistance, we identified 1,025 BDQ-resistant mutants from approximately 5×10^9^ bacteria. The mutation frequency of BDQ-resistant mutants was approximately 2×10^-7^, which is consistent with the frequency observed in previous studies (Andries et al., 2005; Huitric et al., 2010; Nguyen et al., 2018). Our results indicate that *rv0678* mutations may have a low cost *in vitro* and in clinical settings without causing any loss in fitness (Nguyen et al., 2018), which might account for the high rate of BDQ resistance. We randomly selected and analyzed 168/1,025 BDQ-resistant mutants, and in accordance with previous reports (Villellas et al., 2017), our results suggest that *rv0678* mutations are the prominent mechanisms of BDQ resistance. We identified a wide range of mutations within *rv0678*, leading to BDQ resistance, and this improved our understanding of the mechanisms of resistance to BDQ. In addition, our study provided new perspectives on the mechanisms of resistance to BDQ by confirming that *glpK*, a previously undetermined gene, is involved in BDQ resistance.

Rv0678, a MarR-like transcriptional repressor, inhibits the expression of the MmpS5–MmpL5 transport system. MmpL5 forms a complex with MmpS5 to facilitate export of cell wall lipid constituents (Tekaia et al., 1999). Recent studies have shown that the MmpS5–MmpL5 transport system also functions as an efflux pump, and its functional impairment is closely associated with drug resistance (Zhang et al., 2015; Villellas et al., 2017). Rv0678 contains six α helices and two β-strands, which form an L-shape (Radhakrishnan et al., 2014). The shorter side, containing α2, α3, α4, β1, and β2, constructs a DNA-binding domain (Radhakrishnan et al., 2014). The longer side forms a dimerization domain consisting of α1, α5, and α6 (Radhakrishnan et al., 2014). Rv0678 has three conserved amino acids (Arg-82, Asp-88, and Arg-90) that localize to the DNA-binding domain and are critical for protein–DNA interactions (Radhakrishnan et al., 2014). Moreover, Arg-30, Glu-104, Arg-107, Glu-113, Tyr-145, and Tyr-157 are responsible for formation of the dimer (Radhakrishnan et al., 2014). In the present study, most *rv0678* mutations were found either in the DNA-binding domain or in the dimerization domain. Specifically, R90P, 92 codon shifts, and 157 codon shifts in *rv0678*, crucial for protein–DNA interactions and dimer formation, were identified in the present study. Moreover, the *rv0678* insertion and deletion mutations identified in our study caused a frameshift. Therefore, mutants harboring these mutations in *rv0678* may impair the function of either DNA binding or dimerization, leading to loss of function. Our results and those of other groups suggest that mutations in *rv0678* confer resistance to BDQ. Consistently, a spectrum of mutations in *rv0678* in MDR-TB clinical isolates was identified, although most of them were not involved in BDQ resistance (Villellas et al., 2017). Additionally, the high frequency of mutations in *rv0678* was not related to the prior use of BDQ (Villellas et al., 2017). Consistently, our results suggest that *rv0678* mutations are frequent in the presence or absence of BDQ, and BDQ-resistant mutants were selected and enriched after BDQ utilization. A proportion of mutations in *rv0678* identified in the present study were also found in clinical BDQ-resistant isolates (Villellas et al., 2017; Andres et al., 2020; Degiacomi et al., 2020; Nimmo et al., 2020a; Nimmo et al., 2020b), including G198, CC279–280, and TT279–280 deletions and G198, G199, A275, and G439 insertions and G120T substitution. In particular, 67 codon shifts caused by the G198 deletion and G198 and G199 insertions in *rv0678* were the second most common mutations in our BDQ-resistant isolates, indicating that our study contributes to the identification of BDQ-resistant isolates in clinical settings. However, no mutations in *atpE* gene were found in the isolated mutants. We hypothesize that mutations in *atpE* result in relatively high-level resistance to BDQ (10–128-fold MIC); however, the BDQ concentration (0.5 µg/ml, eightfold MIC) used in our study is appropriate for the selection of low-level BDQ-resistant mutants. Consistently, mutations in *rv0678* with a high frequency have been found in clinical isolates, whereas mutations in *atpE* have seldom been identified in clinical isolates (World Health Organization, 2019; Andres et al., 2020).

A previous study found that mutations in *rv0678* cause cross-resistance to clofazimine both *in vitro* and *in vivo* (Hartkoorn et al., 2014; Zhang et al., 2015; Xu et al., 2017). Accordingly, mutants harboring *rv0678* G194A, G269C, G304A, T341C, and T365C mutations were also observed in clofazimine-resistant mutants (Zhang et al., 2015), suggesting that these mutants may be cross-resistant to clofazimine, and our results may provide new insights into clofazimine resistance. Whether the mutants identified in the present study were resistant to clofazimine warrants further investigation.

Although mutations in *atpE*, *rv0678*, and *pepQ* confer major resistance to BDQ, the mechanism of resistance to BDQ for a portion of isolates identified *in vitro* and in clinical settings remains unknown, as they had WT *atpE*, *rv0678*, and *pepQ* (Andres et al., 2020). It is likely that there are other unclarified mechanisms of resistance to BDQ. Here, we identified a new gene, *glpK*, and an insertion mutation in *glpK* (G572 insertion) that resulted in a frame shift and loss of function, leading to BDQ resistance. GlpK, encoded by *rv3696c*, is a critical enzyme for the uptake and metabolism of glycerol and phosphorylates glycerol to glycerol-3-phosphate for the synthesis of glycerophospholipids (Larrouy-Maumus et al., 2013; Whitaker et al., 2020) or for glycolysis and gluconeogenesis (Ehrt and Rhee, 2013). A recent report showed that a frameshift mutation in the 7C homopolymeric tract (HT) sequence (CCCCCCC566-572) of *glpK*, which is equal to the G572 insertion identified in our study, conferred drug tolerance to isoniazid, rifampicin, moxifloxacin, and ethambutol (Safi et al., 2019; Sun et al., 2019). These frameshift mutations in *glpK* lower Mtb growth under certain culture conditions (glycerol-containing culture conditions) but enhance survival after exposure to different anti-TB agents (Safi et al., 2019; Sun et al., 2019). Moreover, *glpK* mutants can revert back to WT *glpK* by introducing additional insertions and deletions in the same *glpK* HT region, thus reversing the slow growth (Safi et al., 2019). The reversible process between frameshift mutations and the WT in the 7C HT sequence of *glpK* conferring reversible drug tolerance during anti-TB therapy facilitates the survival of bacteria *in vivo*, suggesting that similar circumstances may also exist in BDQ resistance. Here, our results showed that mutants harboring the G572 insertion in *glpK* were associated with BDQ resistance (**Table 4**). However, mutants with both G572 insertion in *glpK* and *rv0678* did not increase the MIC to BDQ, suggesting that they have no synergetic or superimposed effects on BDQ resistance (**Table 4**). According to Safi’s study, we hypothesized that the frameshift mutation in *glpK* conferring resistance to BDQ caused reduced Mtb growth (Safi et al., 2019). We found that a large proportion of the isolated mutants formed small colonies with slow growth on 7H10 agar plates (data not shown).

Interestingly, invertible frameshift mutations in HTs is caused by slipped-strand mispairing errors during proliferation, and there is a high-proportioned HT variants in bacteria with DNA mismatch repair defect, including Mtb (Streisinger and Owen, 1985; Cole et al., 1998; Mizrahi and Andersen, 1998; Canceill et al., 1999; Parkhill et al., 2000; Bayliss et al., 2008; Bayliss, 2009), indicating that the poly-G:C and poly-A:T tracts within the Mtb genome might have invertible insertion and deletion mutations during proliferation, such as the G572 insertion in the 7C HT sequence identified in the present study. Moreover, mutants with frameshift mutations in *glpK* in an unstable state have been detected in the sputum of patients with TB (Black et al., 2015; Trauner et al., 2017). Worse still, free glycerol is present in human plasma and Mtb-infected mouse lung tissue, and Safi et al. have demonstrated that the *in vivo* environment offers positive selection for *glpK* mutations (Safi et al., 2019). Therefore, it should bring to the forefront that frameshift mutation in *glpK* caused by mismatch repair deficiency confers BDQ resistance in clinical settings. Together, we revealed a previously undetermined role of *glpK* in low-level BDQ resistance.

In summary, we characterized 168 mutants resistant to BDQ and found that mutations in *rv0678* confer the primary mechanism of BDQ resistance. Importantly, we identified a new gene (*glpK*) involved in BDQ resistance. Further studies are needed to address the role of *glpK* by constructing *glpK* knockout and supplement strains. Our study offers new insights and valuable information that will contribute to rapid identification of BDQ-resistant isolates in clinical settings.

## Data Availability Statement

The original contributions presented in the study are included in the article/**Supplementary Material**. Further inquiries can be directed to the corresponding author.

## Author Contributions

QG and JB performed the experiments. QG, JB, QL, TY, ZhoW, ZhaW, LL, and GZ analyzed the data. QG, JB, and GZ designed the study and wrote the paper. QL, TY, ZhoW, ZhaW, and LL reviewed the paper and supervised the research. All authors contributed to the article and approved the submitted version.

## Funding

This work was supported by the National Key Research and Development Plan (No. 2020YFA0907200), the National Natural Science Foundation of China (Nos. 82170009, 81873958, and 81802058), the Guangdong Scientific and Technological Foundation (Nos. 2019B1515120041 and 2020B1111170014), the Shenzhen Scientific and Technological Foundation (No. KCXFZ202002011007083 and JCYJ20180228162336873), and China Postdoctoral Science Foundation (No. 2020M670085ZX).

## Conflict of Interest

The authors declare that the research was conducted in the absence of any commercial or financial relationships that could be construed as a potential conflict of interest.

## Publisher’s Note

All claims expressed in this article are solely those of the authors and do not necessarily represent those of their affiliated organizations, or those of the publisher, the editors and the reviewers. Any product that may be evaluated in this article, or claim that may be made by its manufacturer, is not guaranteed or endorsed by the publisher.

## Abbreviations

ATP, adenosine triphosphate; BDQ, Bedaquiline; HT, homopolymeric tract; MDR-TB, multidrug-resistant tuberculosis; MIC, minimum inhibitory concentration; Mtb, *Mycobacterium tuberculosis*; OADC, oleic acid, albumin, dextrose, and catalase; TB, tuberculosis; WGS, whole genome sequencing; WT, wild type.
