# Supplementary material for: Whole Genome Sequencing Identifies Novel Mutations Associated With Bedaquiline Resistance in Mycobacterium tuberculosis
Source: Front Cell Infect Microbiol. 2022 May 27;12:807095. doi: 10.3389/fcimb.2022.807095 (PMC9184757; doi:10.3389/fcimb.2022.807095)
Supplement: Supplementary file 1 [file DataSheet_1.pdf]

Table S1. Primers used in the present study.	
**Primers**	**Sequence (5'-3')**
rv0678 F	GAGTTCCAATCATCGCCCTCCG
rv0678 R	CAATCGATAACCTCGGCGCG
glpK F	GAATTCGCGACCCCTTCTCCGG
glpK R	ATTCTCGCCGCCGGGCTAGC
rv2820c F	GGCAGCGGAACCCGTGGCTACGGGC
rv2820c R	CCCTCCACGTGGTACTCCTTCGAGG
